# The association between COVID-19 lockdown and disease severity, quality of life, and mental health in patients with psoriasis: a cross-sectional study in Southwestern China

**DOI:** 10.3389/fpubh.2025.1603623

**Published:** 2025-10-22

**Authors:** Xinyi Shao, Wenyan He, Aijun Chen, Sahil Kapur, Jeffrey Cruz, Ping Wang

**Affiliations:** ^1^Department of Dermatology, The First Affiliated Hospital of Chongqing Medical University, Chongqing, China; ^2^Division of Dermatology, College of Medicine and Life Sciences, University of Toledo, Toledo, OH, United States; ^3^College of Medicine, Central Michigan University, Mount Pleasant, MI, United States

**Keywords:** COVID-19, psoriasis, disease severity, quality of life, mental health, cross-sectional study

## Abstract

**Background:**

Our study aimed to clarify the impact of home quarantine on disease severity, quality of life, and mental health in psoriasis patients through the multidimensional analysis of the status of home quarantine, the severity of psoriasis, quality of life, and depression scores during the COVID-19 pandemic.

**Methods:**

From 2022 to 2023, we conducted telephone follow-up on 963 psoriasis patients. Participants’ demographic characteristics, psoriasis condition, home quarantine duration, quality of life and depression symptom scores were collected. The association between COVID-19 lockdown and patient-reported outcomes were investigated with pearson correlation and Spearman correlation.

**Results:**

A total of 963 participants were recruited, finally 605 participants were enrolled. The mean values of age and disease duration was 43.63 years, 312.35 years, 67.6% were male. Patients with disease-related impaired quality of life (DLQI > 5) accounted for 7.44%. A total of 65 patients had varying degrees of depression symptoms (QIDS-SR16 > 5 points). The result of correlation analysis revealed a positive correlation between BSA and both DLQI and QIDS-SR16 scores (R = 0.27, *p* < 0.001; R = 0.08, *p* < 0.05).

**Conclusion:**

Our results revealed that COVID-19 lockdown had a measurable impact on disease severity, quality of life, and mental health in psoriasis patients. Many individuals experienced varying degrees of symptoms aggravation during the lockdown. The severity of psoriasis was negatively correlated with quality of life and positively correlated with depression symptoms, with older adult patients being particularly vulnerable to depression. These findings highlight the importance for dermatologists to integrate mental health assessment and support into routine psoriasis management.

## 1 Introduction

Psoriasis is a chronic, inflammatory skin disease with a prevalence rate of 0.09–11.43%, which has been increasing over time, affecting approximately 2% of the global population (1). Its main characteristics are scaly plaques on the skin, accompanied by pain and itching, imposing significant physical, psychological, and social burdens on patients (2). The pathogenesis of psoriasis is associated with various genetic and environmental factors (3). Environmental factors such as stress, smoking, alcohol abuse, and obesity also contribute to the development of psoriasis. The immune system plays a crucial role in the pathogenesis of psoriasis, with T-cells considered major players, particularly T-cell-mediated immune inflammatory responses, such as the IL23/IL17 axis, which is thought to play an important role in psoriasis pathophysiology (4). This axis is also involved in the development of many systemic chronic diseases, such as cardiovascular diseases, diabetes, and mental disorders, which are well-recognized comorbidities. Among them, psoriasis is believed to be associated with negative mental health outcomes, including psychosis, suicidal ideation, anxiety, depression, psych (5–10). However, the relationship between them has not been fully established. The COVID-19 pandemic has led to widespread mental health issues, including depression, anxiety disorders, stress, panic, anger, impulsivity, somatic disorders, sleep disorders, mood disorders, post-traumatic stress disorder, and suicidal behavior (11). Studies have shown that the general population experienced psychosocial stress of varying degrees during the COVID-19 pandemic and subsequently developed mental health issues (12, 13). For psoriasis patients, who are more susceptible to negative psychological states, home quarantine appears to exert a greater impact on disease control, quality of life, and mental health; however, no studies have yet explored the impact of COVID-19 home quarantine on disease severity, quality of life, and mental health in psoriasis patients in southwestern of China. Our study aimed to clarify the impact of home quarantine on disease severity, quality of life, and mental health in psoriasis patients through a multidimensional analysis of home quarantine status, psoriasis severity, quality of life, and depression scores during the COVID-19 pandemic.

## 2 Methods

### 2.1 Study design and questionnaires

After reviewing the literature and consulting dermatology and psychiatry experts, a questionnaire with open-ended and closed-ended questions was developed. A telephone follow-up was conducted with 963 psoriasis patients who had visited the dermatology clinic of the First Affiliated Hospital of Chongqing Medical University, where they were informed about the study and provided consent was obtained. After consenting to the survey, they were asked three sections of questions: the first section was about demographic characteristics, psoriasis condition (All patients received brief training from a dermatologist or experienced research fellow experienced in psoriasis clinical data collection including PASI and BSA assessment of patients on how to estimate body surface area (BSA) and then performed self-assessment during the telephone interview. For those unable to perform self-assessment, a video call was arranged to allow virtual estimation), and adherence to treatment; the second section focused on home quarantine duration and COVID-19 infection status; the third section involved quality of life and depression symptom scores.

We used the DLQI to assess the impact of psoriasis on health-related quality of life. The DLQI has been proven to provide reproducible scores with good internal consistency and reliability, making it an important tool in both research and clinical practice for psoriasis patients (14). Depression symptoms were measured using the QIDS-SR16, a reliable and well-validated 16-item self-report questionnaire that evaluates nine domains (sad mood, concentration, self-criticism, suicidal ideation, interest, energy/fatigue, sleep disturbances [insomnia or hypersomnia], appetite/weight disturbance [decrease or increase], and psychomotor agitation or retardation) (15). This study was approved by the ethics committee of The First Affiliated Hospital of Chongqing Medical University, Chongqing, China (Ref no: K2023-080).

### 2.2 Statistical analysis

Continuous variables with normal distribution were expressed as mean ± standard deviation (SD). Student *t*-test were applied to evaluate the difference between groups. If the continuous variables were not subject to normal distribution, the median (interquartile range) was used, and the difference of two groups were evaluated by Mann–Whitney U test. Categorical variables expressed as counts (percentage), compared using Pearson’s chi-square test or Fisher’s exact test. Pearson correlation and Spearman correlation were used for correlation analysis of normal and non-normal data, respectively. All data were analyzed with SPSS 26 (IBM, SPSS Statistics 26), R (version 4.2.1) and Graphpad Prism 8 (GraphPad Software Inc., USA). A *p*-value < 0.05 was considered statistically significant. The Strengthening the Reporting of Observational Studies in Epidemiology (STROBE) statement was used as the basis for reporting cross-sectional studies.

## 3 Results

### 3.1 Demographic characteristics of psoriasis patients

A total of 963 patients were contacted by telephone, of whom 605 were ultimately enrolled in this study. A total of 358 patients were excluded due to incomplete information. The demographic characteristics of patients are shown in Table 1. The mean values of age, age of onset and disease duration was 43.63 years, 31.37 years, 12.35 years, 67.6% were male. A total of 185 patients (30.58%) reported no lesions (BSA = 0), 342 patients (56.53%) had mild psoriasis (BSA 0–3%), and 78 patients (12.9%) had moderate-to-severe psoriasis. Patients with disease-related impaired quality of life (DLQI > 5) accounted for 7.44%. A total of 65 patients had varying degrees of depression symptoms (QIDS-SR16 > 5 points).

**Table 1:** Baseline demographics of psoriasis participants.

Clinical items	Psoriasis patients (*n* = 605)
Gender, male/female	409/196
Age (y), mean ± SD	43.63 ± 15.03
Age of onset (y), mean ± SD	31.37 ± 15.21
Disease duration (y), mean ± SD	12.35 ± 10.74
Body surface area, *n* (%)	
0%	185 (30.57%)
0% <BSA < 3%	342 (56.53%)
3% ≤ BSA ≤ 10%	39 (6.45%)
BSA > 10%	39 (6.45%)
Activity in relation to the COVID-19 lockdown, *n* (%)	
Unlimited	82 (13.55%)
Limited time ≤ 30 days	497 (82.15%)
Limited time > 30 days	26 (4.30%)
DLQI, *n* (%)	
DLQI ≤ 5	560 (92.56%)
DLQI > 5	45 (7.44%)
QIDS-SR16, *n* (%)	
QIDS-SR16 ≤ 5	540 (89.26%)
QIDS-SR16 > 5	65 (10.74%)

### 3.2 Psoriasis affected skin area

**Figure 1:**
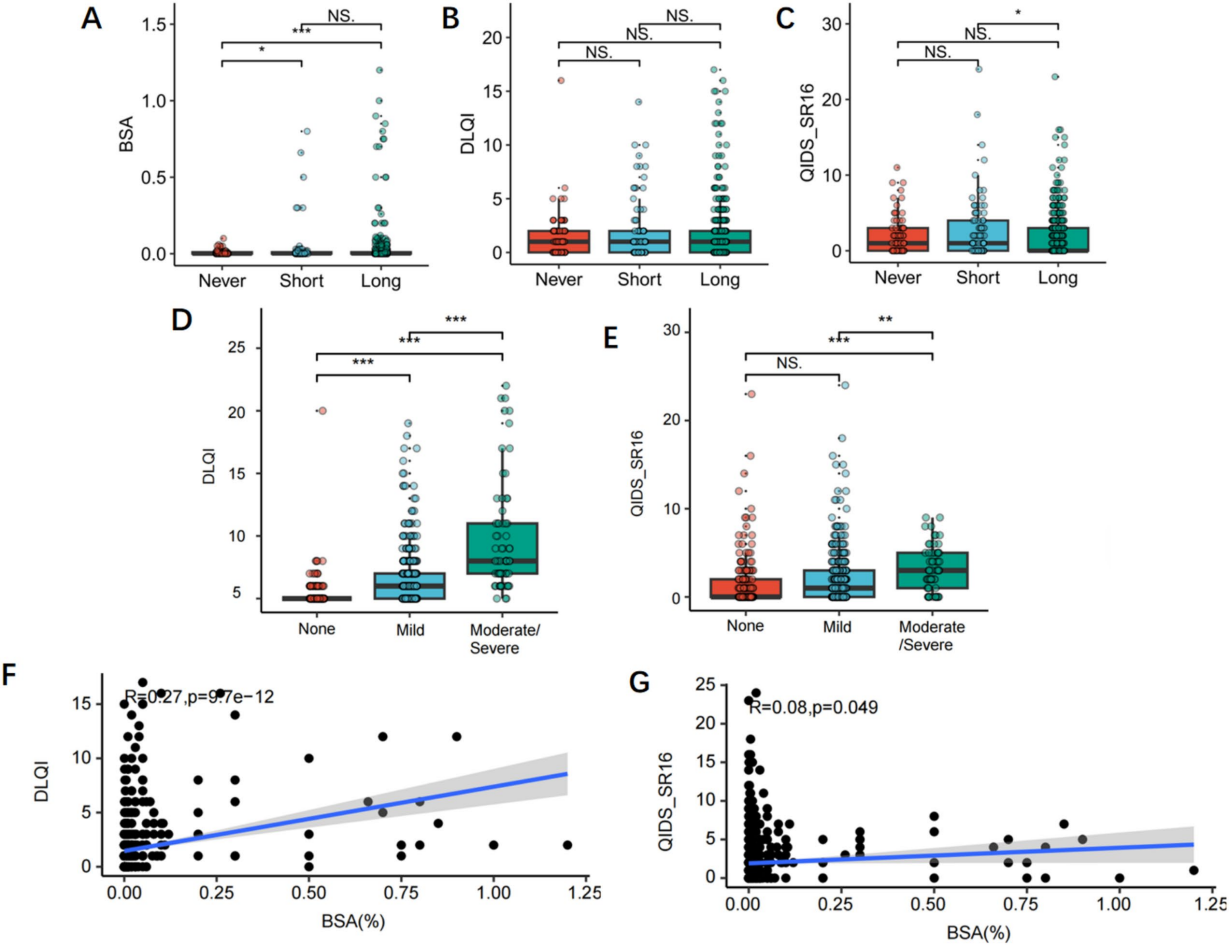
**(A–C)** Subgroup analysis of COVID-19 lockdown duration for BSA area, DLQI score and QIDS-SR16 score. **(D,E)** Subgroup analysis of the severity of psoriasis for DLQI scores and QIDS-SR16. **(D,E)** The correlation between BSA area and DLQI score, and QIDS-SR16 score in psoriasis.

Based on the COVID-19 lockdown duration, we categorized patients into three groups: a non-lockdown group, short-term lockdown group (≤ 30 days), and long-term lockdown group (> 30 days) to f examine associations between lockdown duration and psoriasis BSA. Results indicated that the BSA of patients in the non-lockdown group was significantly lower than those in the short-term lockdown group (*p* < 0.05) and the long-term lockdown group (*p* < 0.001), although there was no significant statistical difference between the short-term and long-term lockdown groups (Figure 1A). DLQI scores and QIDS-SR16 did not differ significantly across the three groups (Figures 1B,C). Additionally, based on the psoriasis-affected skin area, we divided patients into moderate-to-severe psoriasis, mild psoriasis, and no-lesion groups for further analysis of DLQI and QIDS-SR16 scores. We found that DLQI values were significantly higher in mild and moderate-to-severe psoriasis groups compared to the no-lesion group (*p* < 0.01) (Figure 1D). The QIDS-SR16 score was significantly higher in the moderate-to-severe psoriasis group than in both the no-lesion and mild psoriasis groups (*p* < 0.01) (Figure 1E). The result of correlation analysis revealed a positive correlation between BSA and both DLQI and QIDS-SR16 scores (R = 0.27, *p* < 0.001; R = 0.08, *p* < 0.05) (Figures 1F,G).

### 3.3 Impairment of disease-related quality of life

**Figure 2:**
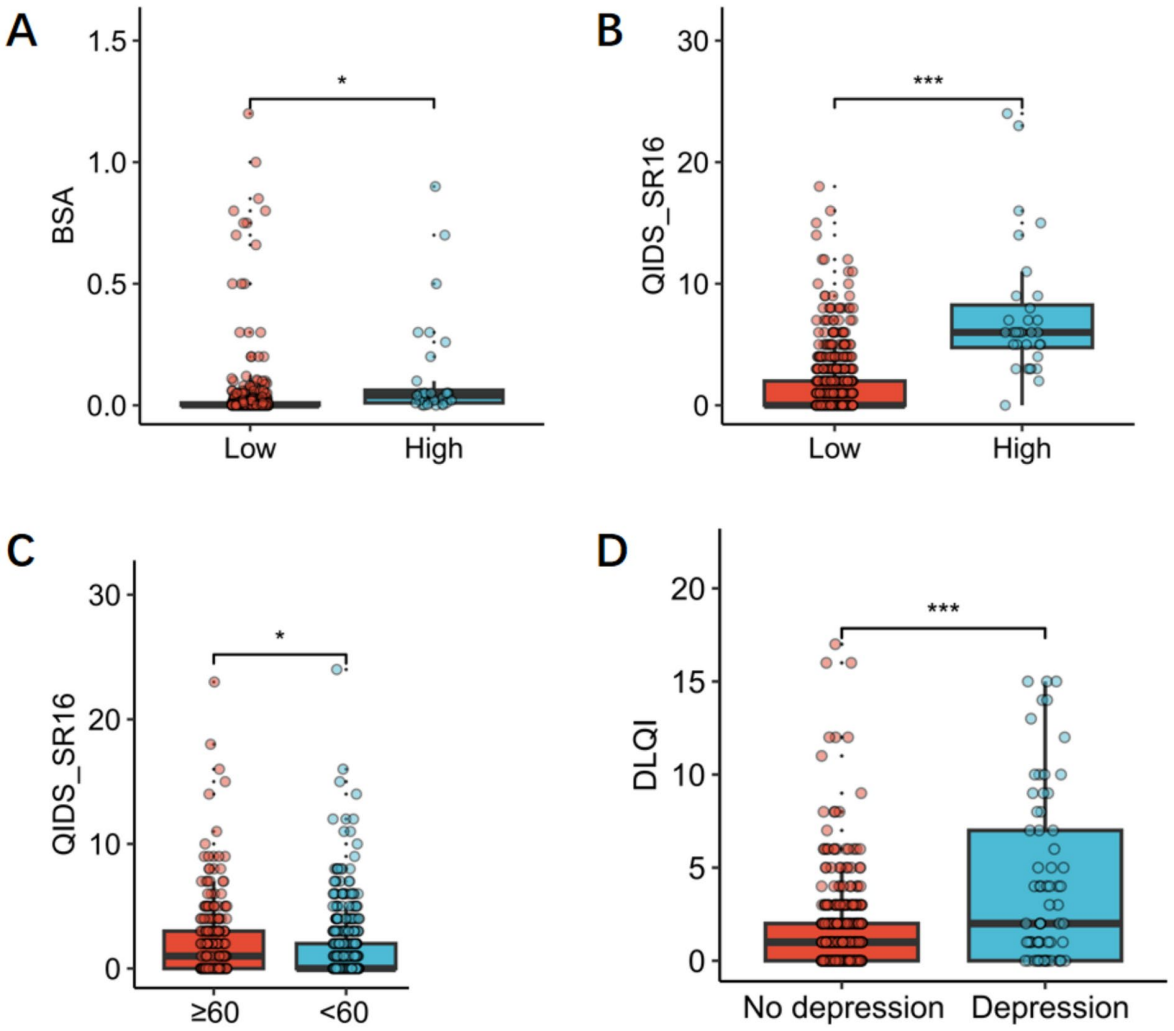
**(A)** BSA area in patients with mild psoriasis and patients with moderate to severe psoriasis. **(B)** QIDS-SR16 scorein patients with mild psoriasis and patients with moderate to severe psoriasis. **(C)** QIDS-SR16 scores in older adult (age ≥ 60) and younger groups (age < 60). **(D)** DLQI scores in the depression group and the non-depression group.

Based on the degree of disease-related quality of life impairment, we categorized patients into groups with no impairment (DLQI ≤ 5) and those with impairment (DLQI > 5) for further analysis. Results showed that impaired group (DLQI score > 5), psoriasis-affected skin area was larger (*p* < 0.05), and depression scores were higher (*p* < 0.001) during the COVID-19 lockdown (Figures 2A,B).

### 3.4 Impairment of depression symptom scores

Based on age, we categorized patients into older adult (age ≥ 60 years) and younger groups (age < 60 years), the analysis revealed that depression symptom scores were significantly higher in the older adult group than in the younger group (*p* < 0.05) (Figure 2C). We also performed subgroup analysis on QIDS-SR16 scores, categorizing patients into two groups: with and without depressive tendencies. Analysis showed that DLQI scores were significantly higher in the depression group than in the non-depression group (*p* < 0.001) (Figure 2D).

## 4 Discussion

In this study, we aimed to investigate whether the COVID-19 lockdown affected clinical symptoms, psoriasis-related quality of life, and mental state in patients with psoriasis in southwest of China. We found a significant correlation between lockdown status and BSA levels of patients; The level of BSA was significantly higher in patients under lockdown compared with without lockdown, and psoriasis severity was positively correlated with DLQI scores. There was also a weak but positive correlation between psoriasis severity and QIDS-SR16 scores. The QIDS-SR16 score was significantly higher in the older adult group than in the younger group.

Psoriasis is a chronic inflammatory skin disease often accompanied by comorbidities such as diabetes, cardiovascular diseases, and depression; shared genetic factors, oxidative stress, overlapping inflammatory processes, and immune dysregulation may underlie their concurrent occurrence. Previous literature has demonstrated that excessive stress can trigger psoriasis and exacerbate the condition (16). Increased sympathetic activity, pro-inflammatory factor release, and hyperactivation of the hypothalamic–pituitary–adrenal (HPA) axis are thought to play significant roles (17, 18). On the other hand, psychosocial functioning in psoriasis patients is often severely impaired, potentially leading to depression, anxiety, and in extreme cases, suicidal ideation or attempts. Despite this, mental health remains under-recognized in routine psoriasis care. For patients particularly vulnerable to psychological issues, the impact of the COVID-19 lockdown cannot be ignored. Several studies have highlighted the negative impact of the COVID-19 lockdown on patients with psoriasis (19–21). Narang et al. (22) found that many patients experienced flares, with financial loss linked to anxiety and depression, while shorter disease duration and a relaxed mental state were protective. Burkauskas et al. (23) similarly reported that decreased income, treatment changes, and depressive symptoms contributed to worsening psoriasis and impaired quality of life, particularly in male patients. In parallel, Pendlebury et al. (23) emphasized the essential role of dermatologists in early recognition and treatment of COVID-related cutaneous manifestations to help reduce complications and improve outcomes. And our previous study found that SARS-CoV-2 infection was associated with COVID-19 vaccination, while booster dose vaccination assisted in lowering the incidence of COVID-19 sequelae. Other major SARS-CoV-2 infection risk factors were female gender, employed individuals, lack of routine protection, severe cases of psoriasis (24). In our study, due to concerns about infection during COVID-19, some patients opted for home lockdown; the BSA was significantly higher in the home lockdown group than in the non-lockdown group, which was associated with medication cessation or modification. In our study, DLQI scores did not differ significantly between short-term, long-term, or non-lockdown psoriasis patients, suggesting that disease burden itself outweighed the incremental effects of lockdown on quality of life. Similar findings were reported by Boch et al. (25), who observed paradoxical stability or improvement in DLQI during lockdown, likely due to reduced social and leisure relevance. By contrast, psychological scores were higher in the short-term lockdown group, reflecting acute stress from sudden restrictions and uncertainty. Over time, patients appeared to adapt, with distress levels attenuating in long-term lockdown—a pattern consistent with other studies linking psoriasis outcomes and psychological stress during the pandemic (23).

Furthermore, our study found that disease-related quality of life and depression symptom scores were significantly higher in mild and moderate-to-severe psoriasis groups compared to the no-lesion group, with positive correlations between BSA and DLQI scores. There was also a weak but positive correlation between psoriasis severity and QIDS-SR16. The results were consistent with previous reports from other regions. These findings suggest that the COVID-19 pandemic imposed an additional burden on psoriasis patients, with disease worsening associated with reduced quality of life and poor mental health. Notably, our study found that depression scores were higher in the older adult group than in the younger group, independent of skin involvement, suggesting that older adult patients may require greater psychological support and treatment.

However, our study has some limitations. First, patients’ self-assessment of mental state and disease flare-up may be inaccurate, as experts did not independently validate these responses. In addition, telephone-based collection may have limited participation for certain individuals. The impact of home isolation and restricted physical activity may also have influenced patients’ mental states, thereby affecting both disease activity and questionnaire responses.

## 5 Conclusion

Our research results revealed the impact of COVID-19 lockdown on disease severity, quality of life, and mental health in psoriasis patients. Many patients experienced varying degrees of psoriasis symptoms during the lockdown. The severity of psoriasis was negatively correlated with quality of life and positively correlated with depression symptoms. Older adult patients were more likely to feel depressed. Dermatologists should focus on patients’ mental health alongside disease management.

## Data Availability

The raw data supporting the conclusions of this article will be made available by the authors, without undue reservation.
